# Inactivation of *Salmonella* using ultrasound in combination with *Litsea cubeba* essential oil nanoemulsion and its bactericidal application on cherry tomatoes

**DOI:** 10.1016/j.ultsonch.2023.106481

**Published:** 2023-06-14

**Authors:** Ruiying Su, Xinyi Guo, Shuai Cheng, Ziruo Zhang, Hui Yang, Jingzi Wang, Luyi Song, Zhande Liu, Yutang Wang, Xin Lü, Chao Shi

**Affiliations:** a College of Food Science and Engineering, Northwest A&F University, Yangling, Shaanxi 712100, China; b College of Horticulture, Northwest A＆F University, Yangling, Shaanxi, China; c School of Science, Xi’an Jiaotong-Liverpool University, Suzhou 215123, China

**Keywords:** *Salmonella*, *Litsea cubeba* essential oil nanoemulsion, Ultrasound, Combined bactericidal effect, Cherry tomatoes

## Abstract

The presence of *Salmonella* in nature poses a significant and unacceptable threat to the human public health domain. In this study, the antibacterial effect and mechanism of ultrasound (US) combined with *Litsea cubeba* essential oil nanoemulsion (LEON) on *Salmonella*. LEON + US treatment has a significant bactericidal effect on *Salmonella*. Reactive oxygen species (ROS), malondialdehyde (MDA) detection, N-phenyl-l-naphthylamine (NPN) uptake and nucleic acid release assays showed that LEON + US exacerbated cell membrane lipid peroxidation and increased the permeability of the cell membrane. The results of field emission scanning electron microscopy (FESEM), transmission electron microscopy (TEM) showed that LEON + US treatment was able to alter cell morphology. It can be observed by flow cytometry (FCM) that LEON + US treatment can cause cell apoptosis. In addition, bacterial counts of cherry tomatoes treated with LEON (0.08 μL/mL) + US (345 W/cm^2^) for 9 min were reduced by 6.50 ± 0.20 log CFU/mL. This study demonstrates that LEON + US treatment can be an effective way to improve the safety of fruits and vegetables in the food industry.

## Highlights

• Ultrasound (US) and *Litsea cubeba* essential oil nanoemulsion (LEON) had a synergistic bactericidal effect.
• LEON + US remarkably disrupted the permeability and integrity of the cell membrane.
• LEON + US altered the morphology and internal microstructure of the cells.
• LEON + US radicalized oxidative stress and lipid peroxidation in cells.
• LEON + US had a good application effect on cherry tomatoes.

## Graphical abstract

**Figure:**
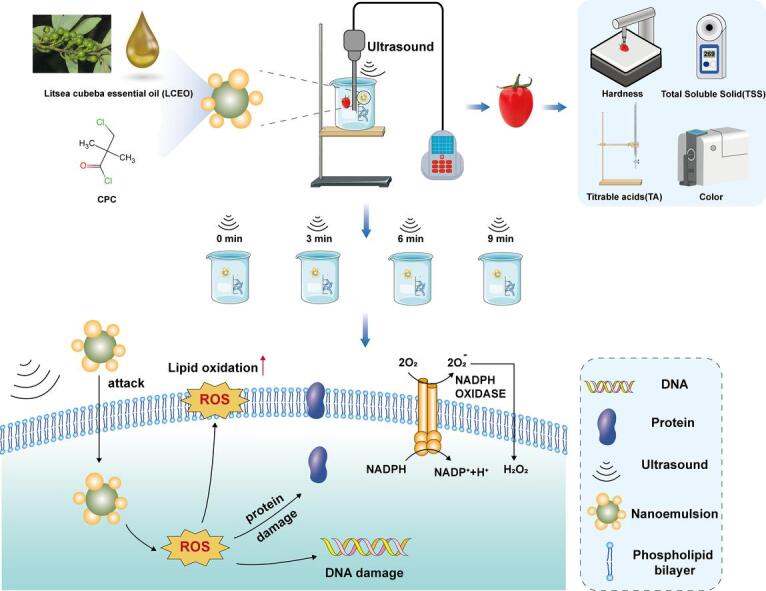

## 1 Introduction

*Salmonella* is a common foodborne pathogen and its infection is a major public health problem worldwide [1]. It has been found that *Salmonella* could contaminate a variety of foods in many ways at any time during planting, harvesting, or processing [2]. Once contaminated, *Salmonella* could enter the fruit through surface cuts or wounds and could survive and multiply in the low pH of cherry tomatoes [3]. Several outbreaks of disease in cherry tomatoes infected by *Salmonella* have been reported in recent years [4]. Therefore, it is important to find a technical method to control *Salmonella* contamination and prevent foodborne disease outbreaks.

Currently, disinfectants, heat treatments, and UV-C are often used to control pathogenic bacteria in the fruit. Hydrogen peroxide, ozonated water, and sodium hypochlorite are used as disinfectants to reduce the bacterial load to maintain the quality and storability of the fruit. However, most of these methods may be harmful to human health and the environment [5]. Conventional heat treatment usually negatively affect the physical properties of the fruit (e.g., texture, flavor, and color) and may loss some valuable nutrients [6]. UV-C has been used in industry as a disinfectant for drinking water and food products such as solids and liquids, but UV-C as a single treatment step is less capable of inactivating a large number of foodborne pathogens on berries [7].

In recent years, as people's living standards have improved, many consumers have sought to use more natural methods to preserve food and control the dangers posed by pathogenic microorganisms in food [8], [9]. *Litsea cubeba* essential oil is mainly extracted from the fresh fruit of *Litsea cubeba* with antibacterial, antioxidant and antiseptic properties [10]. Studies have shown that *Litsea cubeba* essential oil has an inhibitory effect on *Staphylococcus aureus*, *Salmonella* and *Escherichia coli* [11], [12], [13]. However, there are many potential technical challenges in blending essential oils into food as a result of their low water solubility and poor volatility [14], [15]. To overcome these limitations, essential oils could be made into droplets by embedding them in a suitable surfactant. The fine droplets of the nanoemulsion could be effectively absorbed through the biological surface, resulting in efficient and broader biological activity [16]. The nanoemulsion showed great advantages over their counterparts in terms of physical stability and antimicrobial activity, making them more suitable for addition to food products [17].

As a non-thermal technology, US had been widely used in food processing in recent years [18]. US mainly produced a large number of cavitation bubbles through cavitation and mechanical effects to achieve antimicrobial effects [19]. However, a large amount of available data suggested that US treatment alone may not exert sufficient antimicrobial activity to ensure the microbiological safety of food products [20]. Recent studies had shown that US combined with physical or chemical methods was more effective in inactivating bacteria. Guo et al [21] showed that US combined with sodium hypochlorite was effective in controlling *E. coli* in saline. US promoted the destruction of the cytoplasmic membrane and the entry of sodium hypochlorite into the cell, which changed the protein conformation of *E. coli* and ultimately leads to bacterial death. Sagong et al [22] showed that the combination of ultrasonic and organic acid enhanced the combination of organic acid and bacteria, and had an inhibitory effect on *Listeria monocytogenes* in Flammulina velutipes. Currently, the mechanism of inhibition of *Salmonella* by US combined with *Litsea cubeba* essential oil nanoemulsions and its application to foods has not been investigated.

The ultrasonic technique was used to prepare the nanoemulsions of *Litsea cubeba* essential oil, as well as to verify the effects of different ratios of *Litsea cubeba* essential oil and Cetylpyridinium chloride on the average droplet size (Z-average), polydispersity index (PDI) and ζ-potential of the nanoemulsions. At the same time, the bactericidal effect and mechanism of US combined with *Litsea cubeba* essential oil nanoemulsion on *Salmonella* were investigated. It was also applied on cherry tomatoes to explore the effect of this sterilization process on the cleaning of *Salmonella* on the surface, and to observe the effect on the hardness, color, total soluble solids and titratable acids of cherry tomatoes.

## 2 Materials and methods

### 2.1 Reagents and culture conditions of strain

*Litsea cubeba* essential oil (LCEO, CAS: 68855–99-2) was purchased from Sigma-Aldrich (Shanghai, China). Cetylpyridinium chloride (CPC, CAS: 123–03-05) was from Sinopharm Chemical Reagent Co., Ltd. (Shanghai, China). *Salmonella* ATCC 14028 was obtained from the American Type Culture Collection (ATCC; Manassas, VA, USA). Before each assay, *Salmonella* was inoculated on Luria-Bertani (LB) agar and incubated at 37 °C for 12 h to activate the bacteria. They were subsequently incubated in LB broth (12 h, 37 °C). The culture was then diluted in phosphate-buffered saline (PBS) to a wavelength of 600 nm (OD_600 nm_) at an optical density of 0.5 (approximately 10^9^ CFU/mL).

### 2.2 Preparation and characterization of LEON

The surfactant solution was generated by dissolving 500 mg of CPC in distilled water (50 mL), placed on a magnetic stirrer, and stirred at 500 rpm for 5 min. *Litsea cubeba essential oil* (5 mL) was added to the surfactant solution for stirring (500 rpm, 30 min) to form a coarse emulsion. The coarse emulsion was placed on the ultrasonic crusher (20 kHz, Scientz- II D; Ningbo Scientz, Zhejiang, China) to make nanoemulsion (ultrasonic power 450 W, ultrasonic time 9 min, ultrasonic probe diameter 6 mm, ultrasonic pulse 2 s). Before testing, the nanoemulsion was diluted by distilled water at a ratio of 1:100 to eliminate multiple scattering. Z-average, PDI and ζ-potential of LEON were using by Nanolaser particle size analyzer (Malvern Instruments Limited, Worcestershire, UK).

### 2.3 Antimicrobial activity of US combined with LEON treatment

Thirty milliliter of *Salmonella* solution (approximately 10^9^ CFU/mL) and LEON (0.08 μL/mL) were added to a 50 mL sterile cylindrical glass vial, and the probe was inserted 5 mm below the liquid surface. The US power intensities were set at 115, 230 and 345 W/cm^2^ for 3, 6 and 9 min (2 s on; 2 s off). The samples were diluted with PBS and plated onto LB agar and incubated at 37 °C for 24 h.

The experiments were designed as follows: Control sample (without treatment); US samples (115, 230, 345 W/cm^2^); LEON sample (0.08 μL/mL); LEON + US samples, combination of LEON (0.08 μL/mL) and US (115, 230, 345 W/cm^2^). All tests were conducted at 25 °C for 3, 6 or 9 min.

### 2.4 Detection of Reactive oxygen species (ROS)

According to Su et al [23], the fluorescent molecule dichlorodihydrofluorescein diacetate (DCFH-DA; Institute of Biotechnology, Shanghai, China) was used to determine the levels of intracellular ROS of *Salmonella* in LEON, US and combined treatment samples. The bacterial suspensions from each treatment were incubated with DCFH-DA (5 μmol/L) at 37 °C for 10 min. Samples were centrifuged (12,000 × *g*, 10 min) and measured using fluorometric measurements using a multimode microplate reader platform (Spark®; Tecan, Männedorf, Switzerland) with excitation and emission wavelengths of 488 nm and 525 nm, respectively.

### 2.5 Malondialdehyde (MDA) content assay

Determination was performed using the method described by Su et al [23]. The supernatant was collected after centrifugation (8000 × *g*, 10 min) of the samples. The supernatant (300 μL) was mixed with MDA working solution (12,000 μL) at a concentration of 0.67% (*w/v*), boiled at 100 °C for 1 h and then cooled to room temperature. The absorbance of the samples at 450, 532 and 600 nm were measured according to the instructions of the micro-MDA assay kit.

### 2.6 NPN uptake

The NPN uptake follows the method of Qin et al [47], with minor modifications. The treated sample was washed and suspended in PBS. Samples (200 μL/mL) were added to a black 96-well plate, then 1.5 μL NPN (100 mM) was added to each well. Fluorescence was detected using a multimode microplate reader platform (Spark®) with excitation and emission wavelengths of 350 nm and 420 nm, respectively.

### 2.7 Nucleic acid leakage analysis

As described by Li et al [24] with minor modifications. The cell suspensions were centrifuged (8000 × *g*, 10 min, 4 °C) for the collection of supernatants. The content of nucleic acids was measured using a UV–Vis spectrophotometer (UV-2600, Shimadzu, Tokyo, Japan) to determine the absorbance at 260 nm (OD_260 nm_).

### 2.8 Flow cytometry investigation

The effect of US combined with LEON on cell membrane integrity was researched with the method of Lapinska et al [25]. Samples were resuspended in 200 μL of 0.85% (*m/v*) NaCl solution after centrifugation (10,000 × *g*, 4 °C, 2 min). Subsequently, the samples were incubated with 1 μL of an equal volume of SYTO 9 and PI dye mixture in the dark for 15 min. Cell membrane integrity was detected by flow cytometry. (CytoFLEX; Beckman, Brea, CA, USA).

### 2.9 Field emission scanning electron microscope (FESEM) observations

FESEM was performed as described by Song et al [21]. Cells were centrifuged and washed with PBS (5000 × *g*, 10 min, 4 °C). Cells were immobilized with 2.5% (*v/v*) glutaraldehyde and stored overnight at 4 °C. Then, the samples were fixed again with glutaraldehyde for 8 h at 4 °C. After centrifugation (5000 × *g*, 5 min, 4 °C), the samples were eluted by a water–ethanol gradient for 10 min. Finally, the samples were dried and sprayed with gold for observation on FESEM at a magnification of 10,000 × magnification (S-4800; Hitachi, Tokyo, Japan).

### 2.10 Transmission electron microscopy (TEM) observations

According to the method of Cheng et al [20], the intracellular changes were revealed by using TEM. Cell suspensions from each treatment were centrifuged at 5000 × *g* for 10 min at 4 °C after which they were washed twice with PBS. The 2.5% (*v/v*) glutaraldehyde was added to the samples and fixed at 4 °C for 5 h. After centrifugation (5000 × *g*, 10 min, 4 °C), the samples were agar-embedded for a while and then fixed again with glutaraldehyde at 4 °C for 12 h. The samples were embedded in capsules containing white glue and then sectioned for observation by TEM (H-7650; Hitachi, Japan).

### 2.11 Preparation of cherry tomatoes

In this experiment, cherry tomatoes were purchased from local supermarkets in Yangling in terms of size, color, hardness, and absence of damage. Before the start of each experiment, the fruit was washed with distilled water to remove the mud stains and soaked in 75% (*v/v*) ethanol for 10 min. The treated fruit was placed in a laminar airflow to dry. A section (1 × 1 cm) was selected on the surface of the fruit and 50 µL of the bacterial solution (1 × 10^9^ CFU/mL) was inoculated into each of the 5 different locations in the area. After inoculation, the fruit was placed in a sterile station for 1 h to wait for drying. The inoculum volume for the cherry tomatoes was 6.57 ± 0.83 log CFU/mL.

### 2.12 Treatment of cherry tomatoes

After inoculation, the cherry tomatoes were treated by different decontamination processes. Untreated sample: The inoculated cherry tomatoes were soaked in a 500 mL sterile beaker with 300 mL PBS for 3, 6, and 9 min, respectively. LEON treatment sample: LEON was added to 300 mL of bacterial solution at final concentrations of 0.04, 0.06 and 0.08 μL/mL, and inoculated cherry tomatoes were immersed in the solution containing LEON for 3, 6 and 9 min, respectively. Ultrasonic treatment sample: The inoculated cherry tomatoes were soaked in 300 mL of PBS and subjected to ultrasonic treatment at different intensities (115, 230, 345 W/cm^2^) for 3, 6 and 9 min. US combined with LEON treatment sample: LEON was added to 300 mL of PBS (final concentrations of LEON were 0.04, 0.06, 0.08 μL/mL), followed by immediate US treatment (ultrasound intensity of 115, 230, 345 W/cm^2^) for 3, 6, 9 min.

### 2.13 Bacteria quantification on the surface of cherry tomatoes

For exploring the bactericidal of *Salmonella* on the sample surface by different treatments, a section (1 × 1 cm) was cut off into sterile homogenization bags using sterile scissors and homogenized in a homogenizer for 2 min. The bacteria were diluted by PBS and plated onto LB agar at 37 °C for 24 h before counting.

### 2.14 Color analysis

The color of cherry tomatoes was measured using a colorimeter (Minolta Chroma Meter CR-200, Minolta, Osaka, Japan). The instrument was calibrated using white reference tiles. The color parameters consisting of L* (light/dark) and ΔE were evaluated.

### 2.15 Firmness measurements

Texture analysis was performed using a TA.XT2i texture analyzer (Stable Micro Systems Ltd., UK). The texture analyzer was equipped with a 2 mm diameter probe in order to assess the hardness of the whole cherry tomatoes by penetration testing. The testing speed was set at 2 mm/s before and after, the starting force was set at 5 g, and the travel distance was 5 mm. The maximum peak force was measured in hardness and the results were expressed in Newton (N).

### 2.16 Titratable acids (TA) measurement

Titrated acids are determined using NaOH titration, grinding 10 g of tomato and adding it to distilled water to make a sample solution. Accurately aspirate 20 mL of the sample solution, add 3–4 drops of phenolphthalein indicator and titrate with sodium hydroxide standard solution (0.01 N) until the solution is slightly red and does not fade for 30 s. Record the volume of sodium hydroxide consumed.

### 2.17 Total soluble solids (TSS) content

The cherry tomatoes were ground in a mortar and the juice was aspirated, and the TSS content was measured using the juice, which was measured three times for each group using a handheld brix meter (Atago, Tokyo, Japan).

### 2.18 Statistical analysis

Statistical analyses were performed using SPSS software (version 26.0; IBM Corporation, Armonk, NY, USA), and data were expressed as mean ± standard deviation (SD) (n = 3). One-way analysis of variance (ANOVA) was performed, and significance was analyzed using Tukey's test and least significant difference, *P* < 0.05.

## 3 Results

### 3.1 Characterization of nanoemulsion

As shown in Table 1, the average droplet size of the nanoemulsion with a concentration ratio of 1:10 of CPC to *Litsea cubeba* essential oil was 87.20 ± 0.30 nm, which is the smallest droplet size compared with other nanoemulsions. The PDI of this nanoemulsion was 0.20 ± 0.04, there was no difference bewteen three nanoemulsions. The absolute value of zeta potential of nanoemulsion with 1:10 ratio was 61.23 ± 0.20 mV.

**Table 1:** Average droplet size (Z-average), polydispersity index (PDI) and zeta-potential of *litsea cubeba* essential oil nanoemulsion.

Concentration ratio of CPC to essential oil of *Litsea cubeba*	Z-average (nm)	PDI	Zeta potential (mV)
1:1	248.47 ± 0.30^a^	0.25 ± 0.02^a^	55.10 ± 0.50^b^
1:10	87.20 ± 0.30^c^	0.20 ± 0.04^a^	61.23 ± 0.20^a^
1:100	212.63 ± 0.26^b^	0.27 ± 0.03^a^	55.23 ± 0.30^b^

Different lowercase letters indicate statistically significant differences between the means (*P* < 0.05).

### 3.2 Bactericidal effect of US and LEON treatments on *Salmonella*

The amount of initial bacteria was about 8.7 log CFU/mL. From Table 2, it can be seen that as the intensity of US (115, 230 and 345 W/cm^2^) increased, the sterilization effect was increased. The bacteria was decreased by 2.18 ± 0.10, 2.23 ± 0.25 and 2.97 ± 0.14 log CFU/mL after US for 9 min, respectively. With the increase of US (230 W/cm^2^) treatment time, the combined sterilization effect was enhanced and the amount of bacteria decreased by 1.92 ± 0.16, 4.70 ± 0.26, 5.44 ± 0.13 log CFU/mL, respectively. The effect of combined sterilization was greater than that of individual sterilization, and the amount of bacteria decreased by the combined treatment was greater than the sum of the two individual treatments. The amount of *Salmonella* decreased by 8.69 ± 0.02 CFU/mL after 9 min after combined treatment, which was about 4.79 log CFU/mL less than the sum of both.

**Table 2:** Bactericidal effect of ultrasound and LEON treatments on *Salmonella.*

Time /min	US(reduction log CFU/mL)			LEON(reduction log CFU/mL)	LEON + US(reduction log CFU/mL)		
	115 W/cm^2^	230 W/cm^2^	345 W/cm^2^		115 W/cm^2^	230 W/cm^2^	345 W/cm^2^
3	0.17 ± 0.19^cD^	0.24 ± 0.10^bD^	0.25 ± 0.19^cD^	0.67 ± 0.07^bC^	1.25 ± 0.11^cB^	1.92 ± 0.16^cA^	2.18 ± 0.11^cA^
6	0.76 ± 0.05^bDE^	0.51 ± 0.26^bE^	0.82 ± 0.06^bD^	0.88 ± 0.09^bD^	1.89 ± 0.08^bC^	4.70 ± 0.26^bB^	5.54 ± 0.07^bA^
9	2.18 ± 0.10^aD^	2.23 ± 0.25^aD^	2.97 ± 0.14^aC^	0.93 ± 0.13^aE^	5.17 ± 0.13^aB^	5.44 ± 0.13^aB^	8.69 ± 0.02^aA^

Lowercase and uppercase letters represent the differences between the vertical and horizontal rows, respectively.

### 3.3 Effect of different treatments on the intracellular ROS level of *Salmonella*

**Fig. 1:**
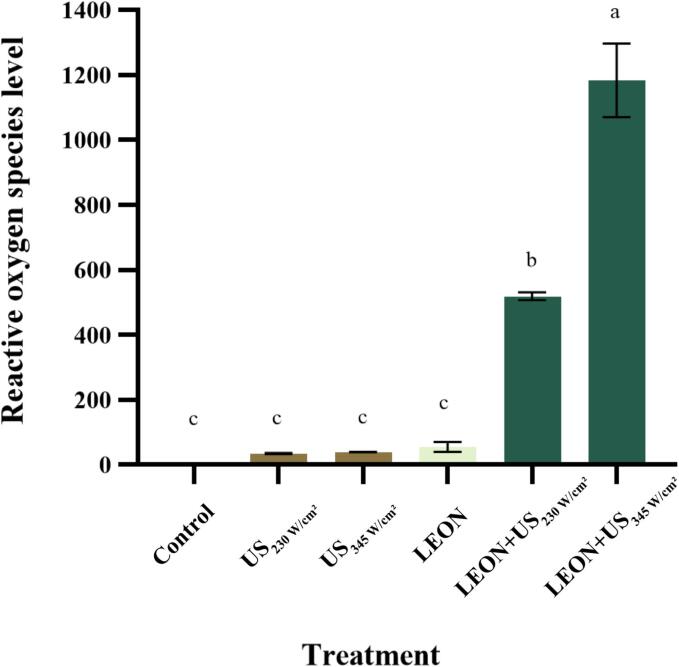
Effect of different treatments on intracellular ROS from *Salmonella*. Different letters indicate statistically significant differences between the treatment groups (*P* < 0.05).

As shown in Fig. 1, the intracellular ROS level in the control was very low and basically undetectable. Intracellular ROS in both ultrasound-alone and nanoemulsion-alone were maintained at a low level with no difference from the control. The level of bacterial ROS was significantly increased (*P* < 0.05) after the LEON + US treatment and increased with the intensity of US. The level of bacterial ROS was significantly increased (*P* < 0.05) after the combined treatment and increased with the intensity of US. The level of bacterial intracellular ROS increased to 519.09 ± 10.29 and 1184.12 ± 92.45 after 6 min of US (230 W/cm^2^ and 345 W/cm^2^) combined with LEON treatment.

### 3.4 Effect of different treatments on extracellular MDA content of *Salmonella*

**Fig. 2:**
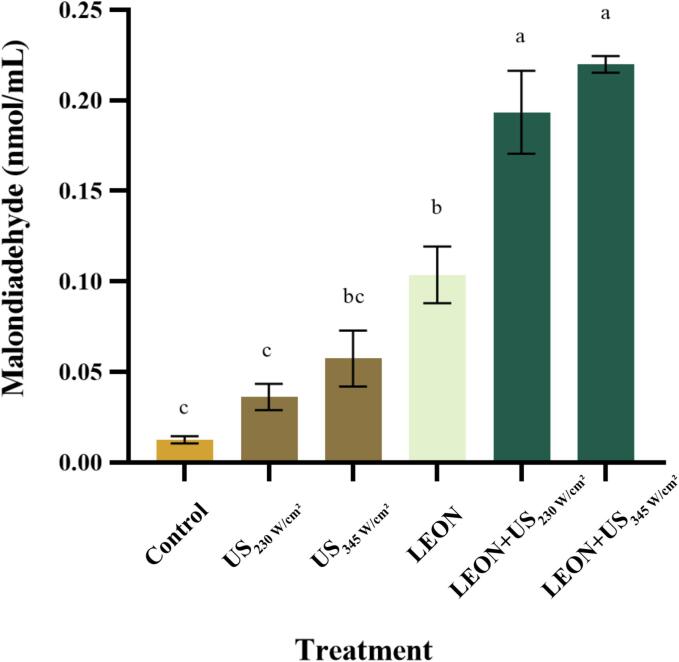
Effect of different treatments on extracellular MDA content from *Salmonella*. Different letters indicate statistically significant differences between the treatment groups (*P* < 0.05).

The effect of different treatments on the MDA content in *Salmonella* was represented in Fig. 2. The extracellular MDA content of the control was 0.013 ± 0.002 nmol/mL. The extracellular MDA content of the ultrasound-alone treatment (230 and 345 W/cm^2^) increased had no difference from the control with increasing US intensity. The extracellular MDA content of LEON-treated bacteria increased to 0.104 ± 0.016 nmol/mL after 6 min. The combined treatment was significantly (*P* < 0.05) different from the control and with increasing US intensity (230 and 345 W/cm^2^) the extracellular MDA content increased to 0.194 ± 0.023 and 0.220 ± 0.005, respectively.

### 3.5 Effect of different treatments on NPN uptake of *Salmonella*

**Fig. 3:**
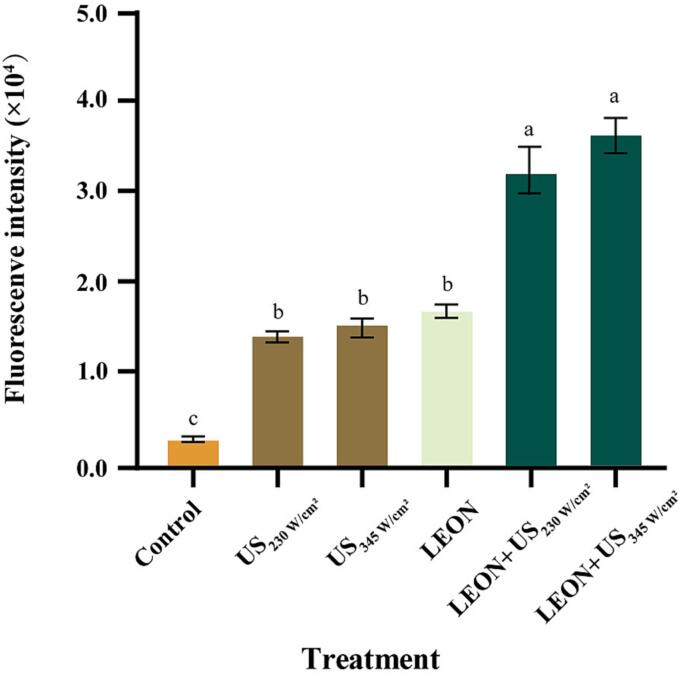
Effect of different treatments on NPN uptake content from *Salmonella*. Different letters indicate statistically significant differences between the treatment groups (*P* < 0.05).

The uptake of NPN by *Salmonella* treated by LEON + US is shown in Fig. 3. Compared with the control group, the fluorescence intensity of NPN increased by 3.04, 3.59 and 4.01 fold after US (230 W/cm^2^ and 345 W/cm^2^) and LEON (0.08 μL/mL) treatments alone, respectively. In addition, the fluorescence intensity of NPN treated by LEON + US was significantly higher than that of US and LEON respectively. As the US intensity increased from 230 W/cm^2^ to 345 W/cm^2^, the fluorescence intensity of NPN also increased.

### 3.6 Effect of different treatments on nucleic acid from *Salmonella*

As shown in Table 3, the content of extracellular nucleic acid was measured by the absorbance value of OD_260nm_. The absorbance value of OD_260nm_ for the control was 0.25 ± 0.01. The nucleic acid content had no difference from the control for 6 min of treatment at 230 W/cm^2^ US intensity, and the increase in extracellular nucleic acid content with increasing US (345 W/cm^2^) intensity reached 0.35 ± 0.01. The absorbance value of OD_260 nm_ for *Salmonella* increased to 0.50 ± 0.02 after 6 min of LEON treatment. The effect of the increase in extracellular nucleic acid content after the combination treatment (230 and 345 W/cm^2^) was 0.60 ± 0.01, 1.14 ± 0.01 for 6 min, respectively.

**Table 3:** Determination of nucleic acids as determined by measuring the absorbance of the aqueous solutions surrounding the bacteria at 260 nm (OD_260 nm_). US: 230 W/cm^2^, 345 W/cm^2^, 6 min, LEON: 0.08 μL/mL, 6 min; and LEON + US treatment for 6 min.

Treatment	OD_260nm_
Control	0.25 ± 0.01^e^
US_230 W/cm_^2^	0.21 ± 0.01^e^
US_345 W/cm_^2^	0.35 ± 0.01^d^
LEON	0.50 ± 0.02^c^
LEON_+_US_230 W/cm_^2^	0.60 ± 0.01^b^
LEON_+_US_345 W/cm_^2^	1.14 ± 0.01^a^

Note: Different letters indicate a significant difference in nucleic acids released from bacteria with different treatments (*P* < 0.05).

### 3.7 Changes in cell membrane integrity of *Salmonella*

**Fig. 4:**
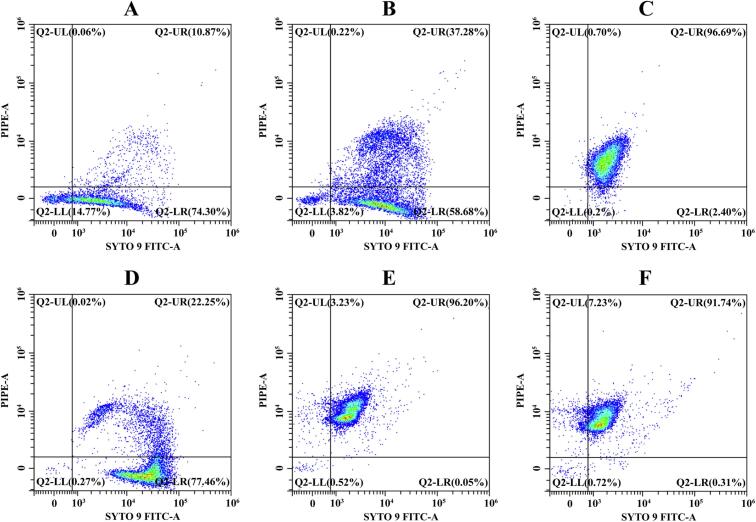
The effects of LEON + US on the membrane integrity of *Salmonella* by flow cytometry. (A) Untreated cells. (B)Treated with ultrasound (230 W/cm^2^). (C) Treated with ultrasound (345 W/cm^2^). (D)Treated with LEON at 0.08 μL/mL. (E)Treated with LEON + US (230 W/cm^2^). (F) Treated with LEON + US (345 W/cm^2^).

The effect of US and LEON on the cell membrane integrity of *Salmonella* was determined by flow cytometry (Fig. 4). Q2-LL, Q2-LR, Q2-UR, Q2-UL represent live unstained, SYTO 9-stained viable bacteria, PI and SYTO 9-stained damaged bacteria, and PI-stained dead bacteria, respectively. The percentage of the number of viable bacteria (Q2-LR) in control was 74.30% (Fig. 4A). As shown in Fig. 4B and C, the number of viable bacteria (Q2-LR) decreased to 58.68% and 2.40%, with increasing US intensity. The LEON treatment elevated the damaged strain (Q2-UR) to 22.25% (Fig. 4D). After the combined treatment of LEON + US, the dead bacteria (Q2-UL) increased to 3.23% and 7.23%, respectively (Fig. 4E and F).

### 3.8 Changes to the surface morphology of *Salmonella*

**Fig. 5:**
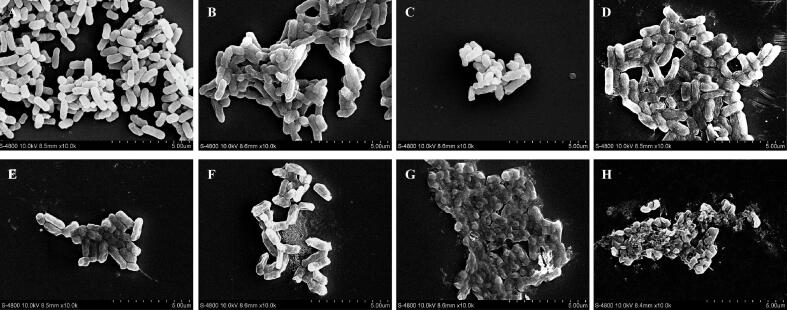
FESEM-based observations (10,000 × magnification) of untreated *Salmonella* (A) and *Salmonella* treated with ultrasound (230 W/cm^2^) for 6 min (B), and *Salmonella* treated with ultrasound (345 W/cm^2^) for 6 min (C), and *Salmonella* treated with ultrasound (345 W/cm^2^) for 9 min (D), and *Salmonella* treated with LEON at 0.08 μL/mL for 6 min (E), and *Salmonella* treated with LEON + US (230 W/cm^2^) for 6 min (F), and *Salmonella* treated with LEON + US (345 W/cm^2^) for 6 min (G), and *Salmonella* treated with LEON + US (345 W/cm^2^) for 9 min (H).

The effect of the LEON, US and combined treatment on cell morphology was observed using FESEM. Cells in the control showed a typical rod-like structure with smooth and dense cell membranes (Fig. 5A). With the increase in treatment time and US intensity, the cell morphology was further disrupted, and the cells were concaved, distorted and wrinkled (Fig. 5B, C, D). *Salmonella* treated with LEON (0.08 μL/mL) was slightly wrinkled with mild surface shrinkage (Fig. 5E). After the treatment with LEON + US (230 W/cm^2^, 6 min), the cell morphology of *Salmonella* collapsed (Fig. 5F), The degree of cell collapse increased after LEON + US (345 W/cm^2^, 9 min) (Fig. 5G). *Salmonella* undergoing LEON + US (345 W/cm^2^, 9 min) treatment ruptured with cell contents appearing to leak and cellular debris being produced (Fig. 5H).

### 3.9 Changes in the internal ultrastructure of *Salmonella*

**Fig. 6:**
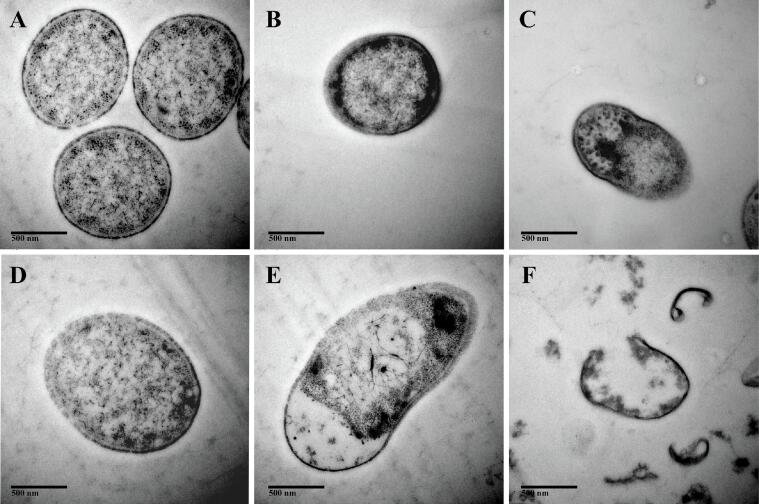
TEM-based observations (40,000 × magnification) of untreated *Salmonella* (A) and *Salmonella* treated with ultrasound (230 W/cm^2^) for 6 min (B), and *Salmonella* treated with ultrasound (345 W/cm^2^) for 9 min (C), and *Salmonella* treated with LEON at 0.08 μL/mL for 6 min (D), and *Salmonella* treated with LEON + US (230 W/cm^2^) for 6 min (E), and *Salmonella* treated with LEON + US (345 W/cm^2^) for 9 min (F).

The effect of different treatments on *Salmonella* internal ultrastructure was explored by using TEM (Fig. 6). The untreated cells were rod-shaped morphology with continuous cell walls and cell membranes, without structural gaps, pores, fissures or interruptions (Fig. 6A). The cell edges were blurred after US treatment (230 W/cm^2^) for 6 min (Fig. 6B). The cells were disrupted after 9 min of US (345 W/cm^2^) treatment and the cell contents started to be released (Fig. 6C). Cells treated with LEON (0.08 μL/mL) were rod-shaped but the cell wall was rough and the cell membrane was blurred (Fig. 6D). LEON + US (230 W/cm^2^, 6 min) treatment resulted in disruption of cell membrane integrity, distortion of cell structure, leakage of internal cellular components. (Fig. 6E). The cell treated with LEON + US (345 W/cm^2^, 9 min) exhibited irreversible cell distortion and piles of cellular debris as seen by Fig. 6F.

### 3.10 Bactericidal effect of *Salmonella* on cherry tomatoes by US and LEON

The initial amount of bacteria on the surface of cherry tomatoes was about 7.0 log CFU/mL. The amount of fruit surface bacteria in the control was reduced by 0.22 ± 0.07, 0.50 ± 0.29 and 0.56 ± 0.04 log CFU/mL at 3, 6 and 9 min, respectively. The bactericidal effect increased with the US intensity, the number of bacteria decreased by 0.56 ± 0.27, 0.82 ± 0.13 and 1.94 ± 0.11 log CFU/mL after 3, 6 and 9 min of US (345 W/cm^2^) treatment alone. The bactericidal effect showed dependence with LEON concentration (0.04, 0.06 and 0.08 μL/mL) decreased by 0.45 ± 0.31, 0.46 ± 0.25, 0.88 ± 0.11 log CFU/mL for 9 min. When LEON (0.04 μL/mL) was combined with US treatment, the bactericidal effect was not as high as the sum of the two individual treatments. With the increase of LEON concentration (0.06 μL/mL) and US intensity (115, 230 and 345 W/cm^2^), the combined bactericidal effect enhanced the amount of bacteria decreased by 1.55 ± 0.27, 2.40 ± 0.23 and 3.38 ± 0.27 log CFU/mL. The strongest bactericidal effect was observed with the combined treatment of LEON (0.08 μL/mL) and US (345 W/cm^2^), reducing the number of bacteria by 1.28 ± 0.28, 2.70 ± 0.14 and 6.50 ± 0.20 log CFU/mL, respectively.

### 3.11 Color analysis

**Fig. 7:**
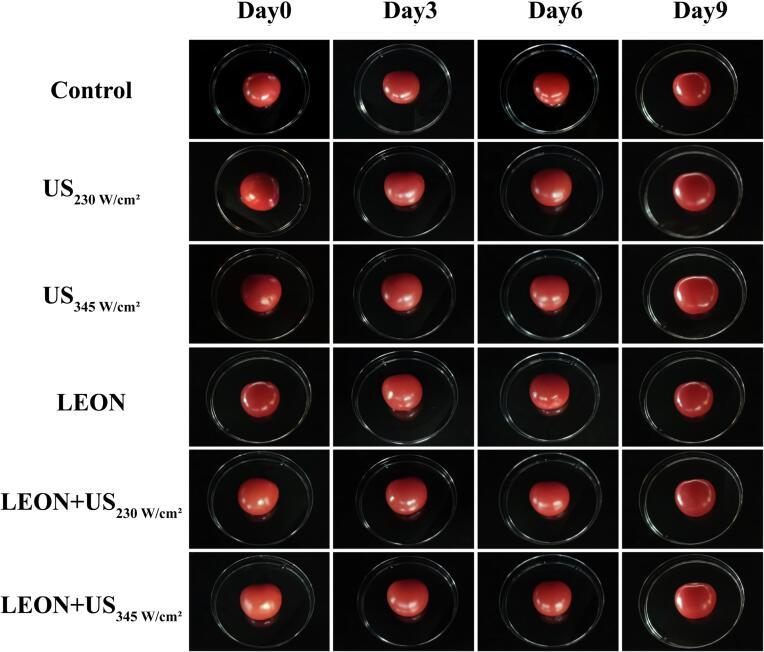
The apparent color of cherry tomatoes under different treatments during storage at 4 °C. US: 230 W/cm^2^, 345 W/cm^2^, 6 min, LEON: 0.08 μL/mL, 6 min; and LEON + US treatment for 6 min, respectively.

The effects of US and LEON on the color of cherry tomatoes were shown in Table 5 and Fig. 7. There was no difference in the brightness values of the control as the storage time increased. US and LEON alone treatment showed no difference in brightness values compared to the control. The brightness values increased for LEON + US (230 W/cm^2^) on the 9th day. The ΔE values of each treatment had no difference from the control with increasing storage time (*P* > 0.05).

### 3.12 Effect of US and LEON treatments on firmness in cherry tomatoes

**Fig. 8:**
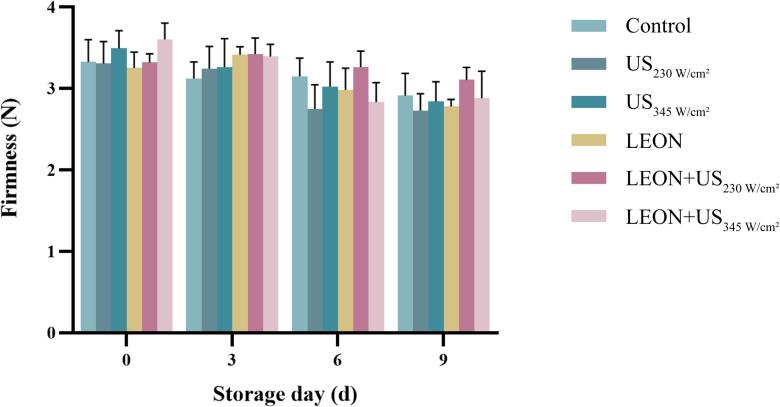
The firmness of cherry tomatoes under different treatments during storage at 4 °C. US: 230 W/cm^2^, 345 W/cm^2^, 6 min, LEON: 0.08 μL/mL, 6 min; and LEON + US treatment for 6 min, respectively.

As shown in Fig. 8, the firmness of the control at 0 d was 3.33 ± 0.22 N. After 9 d of storage, there was no difference in firmness within the control, and no difference in each treatment compared to the control.

### 3.13 Effect of US and LEON treatments on TA in cherry tomatoes

**Fig. 9:**
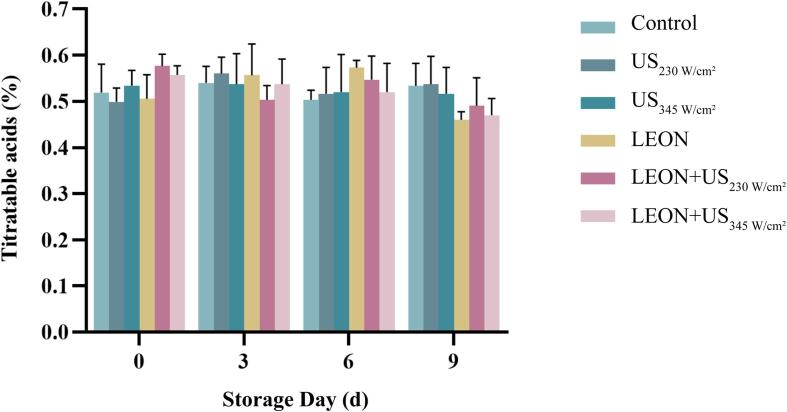
The titratable acidity of cherry tomatoes under different treatments during storage at 4 °C. US: 230 W/cm^2^, 345 W/cm^2^, 6 min, LEON: 0.08 μL/mL, 6 min; and LEON + US treatment for 6 min, respectively.

The effects of US and LEON treatments on TA of cherry tomatoes are given in Fig. 9. The TA content of the control was 0.52 ± 0.07% on 0 d and 0.55% ± 0.04 on 9 d with increasing storage time, with no difference. Throughout the storage period, there was no difference between the treatment and control for the same storage time (*P* > 0.05). At 0,3,6 and 9 d, there was no difference in TA content between US, LEON and LEON + US compared to the control. The TA content was not different between the treatments.

### 3.14 Effect of US and LEON treatments on TSS in cherry tomatoes

**Fig. 10:**
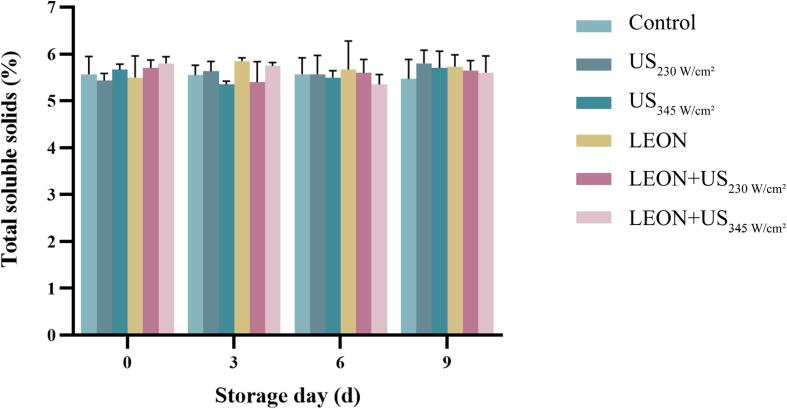
The total soluble solids of cherry tomatoes under different treatments during storage at 4 °C. US: 230 W/cm^2^, 345 W/cm^2^, 6 min, LEON: 0.08 μL/mL, 6 min; and LEON + US treatment for 6 min, respectively.

As shown in Fig. 10, after 0, 3, 6 and 9 d of storage in the control, the total soluble solids (TSS) content in cherry tomatoes was 5.57% ± 0.31, 5.55% ± 0.14, 5.57% ± 0.29, and 5.47% ± 0.34 respectively. TSS content of cherry tomatoes in the control was not affected with the increase of storage time. And there was no effect of US, LEON and LEON + US treatments on TSS of cherry tomatoes compared to the control (*P* > 0.05).

## 4 Discussion

Studies have shown that the smaller the particle size, the more stable the emulsion is to gravity separation or flocculation, and the smaller the PDI, the more stable the emulsion is in a monodisperse state [27]. In this study, the average particle size of LEON made by ultrasonic emulsification was 87.20 ± 0.30 nm, a PDI of 0.20 ± 0.04 and a zeta potential of 61.23 ± 0.20 mV (Table 1), indicating that LEON has small particle size, good dispersion, and high stability. Similarly, Ghazy et al. [26] prepared thyme essential oil nanoemulsion with an average particle size of 143.20 nm using ultrasonic emulsification and Tween 80 as surfactant. Sam et al. [28] showed that the average particle size of sage essential oil nanoemulsion formulated by ultrasonic emulsification using nonionic surface activity was 59.48 nm, indicating that they also prepared good nanoemulsions.

In this study, US (345 W/cm^2^) + LEON (0.08 μL/mL) treatment reduced the number of bacteria by 8.69 log CFU/mL, and the antibacterial effect was significantly better than that of US and LEON treatment alone (Table 2). Similarly, He et al. [38] showed that the number of *E. coli* was reduced by 0.69 and 4.13 log CFU/mL after US (255 W/cm^2^, 9 min) and TEON (0.375 mg/mL) treatment alone, respectively, and the number of bacteria was significantly reduced by 7.42 log CFU/mL after TEON + US treatment. The LEON + US treatment has a stronger inactivation effect in a short, making it more applicable in the food industry to control the microbiological safety of fresh produce.

LEON + US treatment caused an increase in intracellular ROS levels in the bacteria compared to the treatment alone (Fig. 1). Huu et al. [30] showed that the combination of high-frequency US (HFU) and propyl gallate (PG) produced 40% more hydroxyl radicals, an important ROS, than HFU treatment alone after 45 min of *E. coli* treatment. Also, the level of bacterial intracellular ROS was low in *S. aureus* after plasma treatment alone for 2 min, while the combined treatment of US and plasma resulted in a much higher level of ROS production to 1600 [29]. LEON + US treatment generates excessive ROS that attack bacterial cell membranes, disrupting cell membrane permeability and leading to damage to cellular components such as lipids and DNA, and even cell death [28].

MDA is one of the end products of lipid peroxidation, and the content of MDA also indirectly reflects the degree of tissue peroxidative damage [31]. The increase in MDA content in the LEON + US treatment was significantly higher than that in the US and LEON alone (Fig. 2). Yang et al. [32] showed that US (253 W/cm^2^) + citral nanoemulsion (0.3 mg/mL) treatment increased the MDA content of *Salmonella* and was higher than the superimposed effect of two individual treatment.

Combined with the experimental results of ROS, the increase in cell membrane lipid oxidation after combined LEON + US treatment was due to increased membrane permeability caused by excessive ROS production, leading to bacterial death.

NPN is a hydrophobic fluorescent probe that does not penetrate the intact cell membrane and exhibits low fluorescence values in the aqueous environment. In contrast, when the cell membrane structure is disrupted, NPN can diffuse into the hydrophobic environment in the phospholipid bilayer to show fluorescence. The uptake of NPN by Salmonella caused by US + LEON treatment was significantly higher than that by treatment alone (Fig. 3). Similarly, US (253 W/cm^2^) + CLEN (0.4 mg/mL) treatment enhances the uptake of NPN by *Salmonella* [48]. Therefore, the enhanced fluorescence intensity due to LEON + US may be due to the reduced lipid homeostasis of the cell membrane caused by US treatment [49], which increases the permeability of the membrane and thus increases the penetration of LEON into the cell membrane, further destroying the cellular tissue structure.

In the present study, the nucleic acid release of *Salmonella* was significantly increased after LEON + US treatment compared to the US and LEON treatments alone (Table 3). Also, the nucleic acid release of *P. aeruginosa* after US (30 kHz) + slightly acidic electrolytic water (SAW) treatment was significantly higher [45]. The shear force and pressure changes of US during the bubble rupture weakened the cell wall, and the oscillations accompanying the cavitation led to the formation of pores in the cell membrane [46]. The increased contact between LEON and cell membrane contributed to the increase of lipid oxidation of cell membrane, and the combined treatment of LEON + US enhanced the permeability of cell membrane.

A quantitative analysis through flow cytometry showed that low mortality of *Salmonella* in US and LEON treatment alone and significantly higher mortality of *Salmonella* in LEON + US treatment (Fig. 4). The percentage of cell mortality of *S. aureus* exposed to US (400 W) and slightly acidic electrolytic water (SAEW, 2 mg/mL) treatment alone was 6.41 and 23.78% for 10 min, and US + SAEW treatment increased the percentage of cell mortality to 63.74% [33]. The physical effect of US perforates the cell membrane, making it easier for LEON to contact the cell and increasing the interference with the intracellular regulatory mechanism, which leads to cell death.

The surface of *Salmonella* was slightly wrinkled after US and LEON treatment alone, and the combined treatment damaged cell morphology severely, causing cell collapse and leakage of contents (Fig. 5). Huu et al. [30] found that the cell morphology of *E. coli* treated with US and propyl gallate (PG) alone caused some cell damage but most of the cells retained their original morphology. After 10 min of US (1.6 W/cm^2^) + PG (10 mM) treatment, cells appeared to disintegrate and form fragments [34]. In the present study, cell edge blurring after US and LEON treatment alone, and leakage of internal cellular components after LEON + US treatment (Fig. 6). Yang et al. [35] found that US (0.3 W/cm^2^) combined with the net neutral charge peptide TGH2 (125 μg/mL) treated *E. coli* resulted in solute leakage and more severe bacterial morphological damage compared to treatment alone. LEON + US treatment increased intracellular ROS levels, increased lipid oxidation, and disrupted cell membrane integrity, leading to leakage of contents and causing changes in cell morphology, resulting in irreversible cellular damage.

The LEON + US treatment significantly reduced the amount of *Salmonella* on the surface of cherry tomatoes compared to the US and LEON treatment alone (Table 4). Similarly, Zhang et al. [36] found that the combined treatment of citral (10 mM) and US (20 kHz) with *E. coli* on the surface of blueberries reduced the bacterial load by 5.23 log CFU/g after 15 min. The bactericidal effect of the combined treatment was higher than the treatment alone. Millan-Sango et al. [37] found that the combined treatment of US (26 kHz, 200 W) and oregano essential oil (0.025% *v/v*) on lettuce for 5 min reduced the number of *E. coli* by 3.87 ± 0.28 CFU/cm^2^. There were significant differences compared to the samples treated without oregano essential oil.

**Table 4:** Bactericidal effect of ultrasound and LEON treatments of *Salmonella* on cherry tomatoes.

Time /min	Control	US (reduction log CFU/mL)				LEON (reduction log CFU/mL)				LEON + US (reduction log CFU/mL)							
									0.04 μL/mL			0.06 μL/mL			0.08 μL/mL		
		115 W/cm^2^	230 W/cm^2^	345 W/cm^2^			0.04 μL/mL	0.06 μL/mL	0.08 μL/mL	230 W/cm^2^	345 W/cm^2^	115 W/cm^2^	230 W/cm^2^	345 W/cm^2^	115 W/cm^2^	230 W/cm^2^	345 W/cm^2^
3	0.22 ± 0.07^bDE^	0.45 ± 0.27^bCDE^	0.47 ± 0.10^cCDE^	0.56 ± 0.27^bCD^		0.19 ± 0.07^aDE^	0.17 ± 0.10^bDE^	0.70 ± 0.08^bBC^	0.23 ± 0.28^bCDE^	0.09 ± 0.09^cE^	0.17 ± 0.09^cDE^	0.17 ± 0.09^cDE^	0.34 ± 0.12^cCDE^	0.67 ± 0.19^cBC^	0.40 ± 0.27^bCDE^	1.04 ± 0.11^cAB^	1.28 ± 0.28^cA^
6	0.50 ± 0.29^abEFG^	0.51 ± 0.20^bEFG^	0.77 ± 0.05^bE^	0.82 ± 0.13^bDE^		0.21 ± 0.09^aG^	0.23 ± 0.10^abFG^	0.76 ± 0.12^abE^	0.42 ± 0.21^bEFG^	1.37 ± 0.23^bBC^	1.22 ± 0.12^bCD^	0.54 ± 0.20^bEFG^	1.40 ± 0.23^bBC^	1.70 ± 0.14^bB^	0.62 ± 0.19^bEF^	2.41 ± 0.23^bA^	2.70 ± 0.14^bA^
9	0.56 ± 0.04^aGH^	0.95 ± 0.10^aG^	1.88 ± 0.09^aDE^	1.94 ± 0.11^aDE^		0.45 ± 0.31^aH^	0.46 ± 0.25^aH^	0.88 ± 0.11^aG^	1.39 ± 0.12^aF^	1.97 ± 0.09^aCDE^	2.40 ± 0.23^aC^	1.55 ± 0.27^aEF^	2.40 ± 0.23^aC^	3.38 ± 0.27^aB^	2.07 ± 0.38^aCD^	3.80 ± 0.15^aB^	6.50 ± 0.20^aA^

Lowercase and uppercase letters indicate significant differences between different days within the same treatment and between different treatment groups for the same day, respectively.

There was no difference in L* values of cherry tomatoes in each treatment group compared to the control, but L* values were elevated in the combined treatment at 9 day of storage (Table 5). It may be due to the release of anthocyanins in US treatment at specific time and power levels that may cause an increase in brightness values [28]. There was no difference between US, LEON and LEON + US treatments on ΔE, TA, TSS content and firmness of cherry tomatoes compared to the control (Table 5 and Fig. 9, Fig. 10). Ding et al. [39] found no effect of US in combination with SAEW on ΔE, TA and TSS content in cherry tomatoes. Irazoqui et al. [40] also demonstrated that the combined treatment of US and NaClO during storage had no effect on the firmness of lettuce.

**Table 5:** Effect of different treatments on the color attributes of cherry tomatoes.

Storage time/d	Treatment					
	Control	US		LEON	LEON + US	
		230 W/cm^2^	345 W/cm^2^		230 W/cm^2^	345 W/cm^2^
L*						
t = 0	34.8 ± 0.54^aAB^	34.2 ± 0.30^aB^	35.9 ± 0.47^aAB^	35.5 ± 0.37^aB^	36.4 ± 0.17^aA^	35.0 ± 0.08^abAB^
t = 3	34.9 ± 0.14^aAB^	34.2 ± 0.10^aB^	35.2 ± 0.42^aAB^	34.4 ± 0.06^aB^	35.9 ± 0.11^aA^	35.1 ± 0.28^abAB^
t = 6	34.6 ± 0.27^aAB^	34.1 ± 0.52^aB^	35.2 ± 0.22^aAB^	34.2 ± 0.48^aAB^	35.6 ± 0.21^aA^	35.7 ± 0.35^aA^
t = 9	34.1 ± 0.16^aB^	34.4 ± 0.38^aAB^	35.2 ± 0.02^aAB^	34.1 ± 0.69^aB^	35.6 ± 0.37^aA^	34.1 ± 0.15^bB^
ΔE						
t = 0	4.09 ± 0.28^aA^	3.84 ± 0.16^aA^	3.58 ± 0.45^aA^	3.34 ± 0.48^aA^	3.28 ± 0.22^aA^	3.25 ± 0.32^aA^
t = 3	3.89 ± 0.17^aA^	3.92 ± 0.40^aA^	3.86 ± 0.55^aA^	4.03 ± 0.71^aA^	3.79 ± 0.24^aA^	3.73 ± 0.32^aA^
t = 6	3.74 ± 1.16^aA^	3.84 ± 0.22^aA^	3.71 ± 0.59^aA^	3.78 ± 0.46^aA^	3.67 ± 0.28^aA^	3.49 ± 0.17^aA^
t = 9	4.07 ± 0.28^aA^	3.99 ± 0.45^aA^	3.86 ± 0.54^aA^	3.72 ± 0.38^aA^	3.73 ± 0.08^aA^	3.84 ± 0.40^aA^

Lowercase and uppercase letters indicate significant differences between different days within the same treatment and between different treatment groups for the same day, respectively.

The fresh fruit and vegetable industry often use sodium hypochlorite for inhibition [41]. The use of sodium hypochlorite for washing cherry tomatoes can effectively reduce the cross-infection of pathogenic microorganisms in the washing water and also remove pathogens from the surface of fruits [42]. However, sodium hypochlorite reacts with organic matter in the wash water, producing by-products that are considered carcinogenic [43]. This poses a potential threat to the safety and health of consumers. Tap water is often used to wash fruits and vegetables at home, and soaking cantaloupe in tap water reduced the amount of *Salmonella* by 0.7 log CFU/g for 60 s. Although washing fresh produce in tap water removes debris or dirt, it is not effective in removing microorganisms and can lead to cross-contamination of food surfaces, utensils, and other foods [44].

In general, this study showed that LEON combination with US for a short period of time was considered a better antibacterial method to maintain the quality of sainfoin.

## 5 Conclusion

In the present study, the LEON made by ultrasonic emulsification was a well dispersed nanoemulsion. The inhibition effect of LEON + US was significantly more effective compared to the US and LEON treatment alone, with a combined effect on inactivating bacteria. LEON + US treatment leads to increased ROS levels, MDA content, NPN uptake, leakage of cellular components, disruption of cell morphology and cell membranes, which in turn leads to *Salmonella* death. Meanwhile, LEON + US treatment can effectively control *Salmonella* from the surface of the cherry tomatoes, while the treatment did not change the surface color, firmness, TA and TSS content. In conclusion, LEON + US is an effective method for cleaning cherry tomatoes, and this study provides a theoretical possibility to apply this bactericidal method in the food industry in order to improve the safety of fresh produce.

## CRediT authorship contribution statement

**Ruiying Su:** Conceptualization, Investigation, Formal analysis, Project administration, Writing – original draft, Writing – review & editing. **Xinyi Guo:** Resources, Investigation, Data curation, Visualization. **Shuai Cheng:** Resources, Data curation, Investigation. **Ziruo Zhang:** Data curation, Resources, Methodology. **Hui Yang:** Visualization, Formal analysis, Resources. **Jingzi Wang:** Methodology, Supervision, Software. **Luyi Song:** Visualization, Formal analysis, Validation. **Zhande Liu:** Visualization, Formal analysis, Validation. **Yutang Wang:** Methodology, Resources, Supervision. **Xin Lü:** Supervision, Project administration. **Chao Shi:** Project administration, Supervision, Funding acquisition.

## Declaration of Competing Interest

The authors declare that they have no known competing financial interests or personal relationships that could have appeared to influence the work reported in this paper.
